# Automated quantification of posterior vitreous inflammation: optical coherence tomography scan number requirements

**DOI:** 10.1038/s41598-021-82786-0

**Published:** 2021-02-08

**Authors:** Jan Henrik Terheyden, Giovanni Ometto, Giovanni Montesano, Maximilian W. M. Wintergerst, Magdalena Langner, Xiaoxuan Liu, Pearse A. Keane, David P. Crabb, Alastair K. Denniston, Robert P. Finger

**Affiliations:** 1 Department of Ophthalmology, University Hospital Bonn, 53127 Bonn, Germany; 2 Division of Optometry and Visual Science, City, University of London, London, UK; 3 Academic Unit of Ophthalmology, Institute of Inflammation and Ageing, University of Birmingham, Birmingham, UK; 4 Department of Ophthalmology, University Hospitals Birmingham NHS Foundation Trust, Birmingham, UK; 5 NIHR Biomedical Research Centre At Moorfields Eye Hospital and UCL Institute of Ophthalmology, London, UK

**Keywords:** Diagnostic markers, Diagnostic markers

## Abstract

Quantifying intraocular inflammation is crucial in managing uveitis patients. We assessed the minimum B-scan density for reliable automated vitreous intensity (VI) assessment, using a novel approach based on optical coherence tomography (OCT). OCT volume scans centered on the macula were retrospectively collected in patients with uveitis. Nine B-scans per volume scan at fixed locations were automatically analyzed. The following B-scan selections were compared against the average score of 9 B-scans per volume scan as a reference standard: 1/3/5/7 central scans (1c/3c/5c/7c), 3 widely distributed scans (3w). Image data of 49 patients (31 females) were included. The median VI was 0.029 (IQR: 0.032). The intra-class-correlation coefficient of the VI across the 9 B-scans was 0.923. The median difference from the reference standard ranged between 0.001 (7c) and 0.006 (1c). It was significantly lower for scan selection 3w than 5c, p(adjusted) = 0.022, and lower for selection 7c than 3w, p(adjusted) = 0.003. The scan selections 7c and 3w showed the two highest areas under the receiver operating curve (0.985 and 0.965, respectively). Three widely distributed B-scans are sufficient to quantify VI reliably. Highest reliability was achieved using 7 central B-scans. Automated quantification of VI in uveitis is reliable and requires only few OCT B-scans.

## Introduction

Uveitis is a common inflammatory disease of the eye, accounting for 5–10% of visual impairment worldwide^1,2^. The disease affects the vascular layer (consisting of iris, ciliary body and choroid) of people who are frequently of working age^1,3^. Quantification of intraocular inflammation is crucial in managing patients with uveitis. To date the quantification of intraocular inflammation is mostly done semi-quantitatively by subjective clinical evaluation, which comes with a range of limitations common to subjective ratings^3–5^. Thus, several approaches have been developed to quantify vitreous intensity (VI) more objectively^6–9^. This includes quantification of vitreous inflammation based on optical coherence tomography (OCT) scans^8–12^.

The developed algorithm for an automatic assessment of vitreous inflammation is based on the measurement of hyperreflective spots within the posterior vitreous included on macular OCT scans. As this parameter alone is prone to artefacts due to media opacities, a score relative to the retinal pigment epithelium has been established in previous studies and evaluated against the reference standard of the Standardization of Uveitis Nomenclature (SUN) clinical grading of vitreous haze^8–13^.

The application of an OCT-based, automated algorithm for quantification of vitreous inflammation requires manual selection and a certain amount of manual post-processing steps of scans. For this reason, the number of scans should be limited to the minimum amount required for reliable quantification of VI to facilitate future employment in clinical routine and randomized controlled clinical trials. These applications include a potential use of the OCT-based parameter as a biomarker for therapeutic decisions, follow-up intervals and as a clinical trial endpoint. Thus, we assessed the minimum required number of B-scans to reliably quantify vitreous inflammation in this study.

## Results

Current image data of 49 eyes of 49 patients (31 females, 18 males) examined at a tertiary referral centre were included. Uveitis was classified as intermediate in 8 eyes, posterior in 33 eyes and panuveitis in 8 eyes. Mean age at examination was 70 ± 12 years; mean logMAR BCVA at examination was 0.5 ± 0.3 and 44 eyes were pseudophakic.

**Figure 1:**
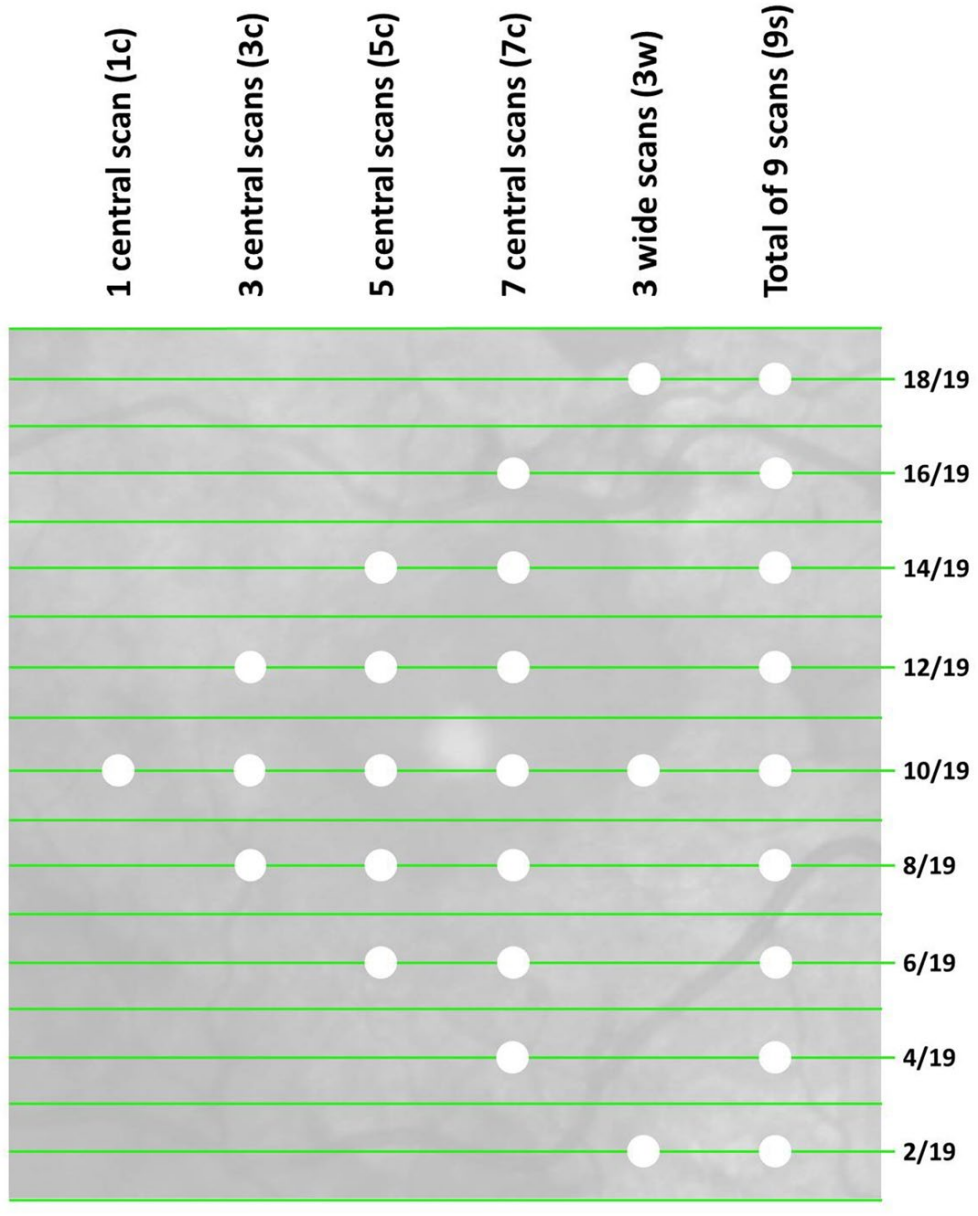
Illustration of an infrared image linked to an OCT volume scan that consists of 19 B-scans. The white dots indicate which scans (green lines) have been included in the different sub selections of B-scans (columns).

The mean distance between two B-scans was 243 ± 8 µm (Fig. 1, individual B-scans are represented by green lines). Across all B-scans, the median VI was 0.029 (interquartile range: 0.032), ranging from 0.0026 to 0.394. The mean VI per eye did not differ significantly between phakic and pseudophakic eyes (*P* = 0.919). The intra-class correlation coefficient of the VI values across the 9 B-scans was 0.923 (95% confidence interval 0.886 – 0.952), indicating high agreement between VI values.

Smaller median differences indicate less variation from the chosen reference standard (i.e. the mean VI value from 9 B-scans, Fig. 1). Table 1 shows that the median differences between the reference standard and the average values of scan selections (1 central scan, 1c; 3 central scans, 3c; 5 central scans, 5c; 7 central scans, 7c and 3 widely distributed scans, 3w) were noticeably different. For instance, the difference from the total VI average in 9 B-scans was smaller in the sub selection 3w compared to 5c (Holm-Bonferroni adjusted *P* = 0.022). It was also smaller in the sub selection 7c compared to 3w (Holm-Bonferroni adjusted *P* = 0.003).

**Table 1:** Deviation between the reference standard (VI means of 9 B-scans) and average VI values from the sub selections of B-scans and respective limits of agreement.

Scan sub selection	Median VI difference from reference standard (interquartile range)	Limits of agreement compared to reference standard
1 central scan (1c)	0.006 (0.009)	[− 0.039;0.037]
3 central scans (3c)	0.005 (0.011)	[− 0.033;0.032]
5 central scans (5c)	0.004 (0.009)	[− 0.028;0.026]
7 central scans (7c)	0.001 (0.004)	[− 0.009;0.009]
3 wide scans (3w)	0.003 (0.005)	[− 0.014;0.013]

VI, vitreous intensity.

Linear regression analysis revealed no significant associations between the two axes on Bland–Altman plots with each other, comparing the reference standard with the VI scores from individual scan selections (comparators: 1c, p = 0.907; 3c, p = 0.120; 5c, p = 0.172; 7c, p = 0.604; 3w, p = 0.243).

**Figure 2:**
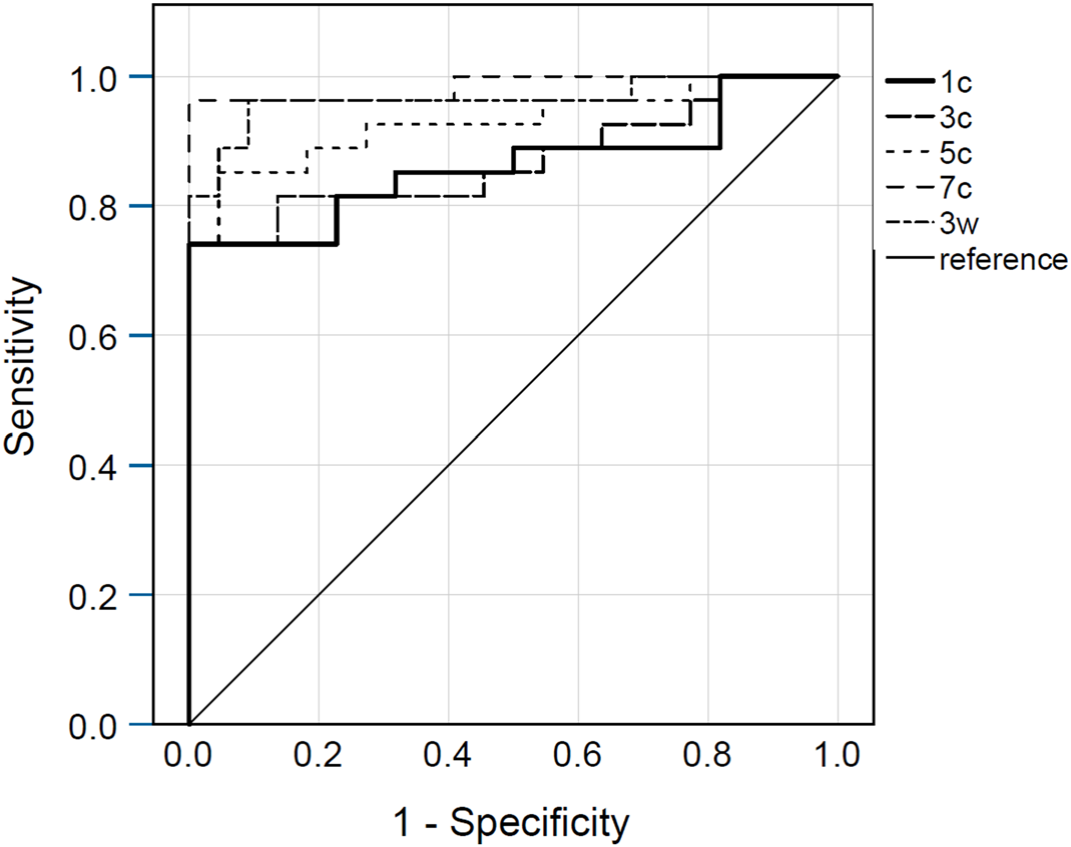
Receiver operating curves of the different scan sub selections. The state variable was the dataset’s median. 1c, 1 central scan; 3c, 3 central scans; 5c, 5 central scans; 7c, 7 central scans; 3w, 3 wide scans.

All area under the curve (AUC) values from ROC analysis were > 0.8 (Table 2), indicating high sensitivity and specificity for the detection of values larger than the dataset’s median, i.e. detection of eyes with statistically “higher inflammation” in contrast to eyes with “lower inflammation”. The scan sub selections 7c and 3w achieved the highest sensitivity and specificity values (Fig. 2). We achieved similar results using a comparison with the dataset’s upper and lower quartile as state variables (data not shown).

**Table 2:** Area under the curve values for the detection of values larger than the dataset’s median, per scan sub selection.

Scan sub selection	AUC [95% CI]
1 central scan (1c)	0.862 [0.753; 0.971]
3 central scans (3c)	0.870 [0.767; 0.974]
5 central scans (5c)	0.929 [0.855; 1.0]
7 central scans (7c)	0.985 [0.954; 1.0]
3 wide scans (3w)	0.965 [0.912:.1.0]

AUC, area under the curve; CI, confidence interval.

## Discussion

The results indicate that Vitreous/RPE-relative intensity is consistent across OCT scans in individuals with uveitis. Averaging the VI of several B-scans within one OCT volume scan further improved measurement reliability because it reduces the influence of local structural alterations. VI calculation from three widely distributed B-scans (average distance 1944 µm) achieves comparable results with VI calculation from nine equally distributed B-Scans (average distance 486 µm) and allows for sufficient discrimination of different levels of inflammation.

Our results indicate that less dense scan patterns compare well to more dense scans in the quantification of VI. As the biomarker itself as well as the measurement of vitreous inflammation using OCT are relatively novel, no data comparing different scan densities for this purpose are available so far. However, different OCT scan patterns of the macula have been compared in the literature with respect to detection of retinal biomarkers such as the presence of intraretinal fluid and quantification of retinal layer thicknesses. Sayanagi and colleagues did not find a significant difference in retinal thickness measurements of patients with macular diseases between a dense OCT volume scan and a radial scan consisting of 6 lines^14^. Other groups confirmed that retinal thickness can be measured almost as reliably with low B-scan density scans compared to high density scans in individuals with defined macular diseases such as diabetic macular oedema^15,16^, age-related macular degeneration^17^ and retinal vein occlusion^18^. Different studies showed the detection of fluid to be almost as sensitive in scan patterns only 25–50% as dense as the respective reference standard in age-related macular degeneration, diabetic macular oedema, retinal vein occlusion and other retinal diseases^17–23^.

Our main result that a pattern with a smaller number of OCT B-scans-based measurements is similarly sensitive as a denser reference standard is thus consistent with findings reported in the literature. The scan sub selection “3w” including a central B-scan and two peripheral B-scans was superior to a single central scan, 3 central scans and 5 central scans. Reliability can be increased with 7 B-scans, i.e. the difference from the reference standard was significantly lower, but the relevance of this small decrease in mean difference is unclear and needs to be considered against the increased workload.

Of note is that the minimum scan density that can be recommended for the measurement of VI is lower than the one recommended for use in retinal diseases to assess retinal thickness or presence of macular oedema reported in the literature. The minimum number of B-scans required for these purposes varies between five and 32 scans^17–23^. The diffuse nature of the signal in the vitreous cavity in inflammatory diseases in contrast to clearly locatable pathologies in only a small part of the retina in retinal diseases might be a potential explanation for this. However, the impact of local heterogeneity in vitreous haze or accumulation of inflammatory cells (e.g. snowballs) on the OCT-based parameter and its changes with eye movement require further investigation.

The strengths of our study include a standardized imaging protocol for all participants, a relatively homogenous sample consisting of only uveitis patients (excluding anterior uveitis) and the use of a previously developed algorithm which is already clinically validated. Limitations include the relatively small sample size with only a limited number of B-scans per subject available, the use of only one device (Spectralis, Heidelberg Engineering), the relatively high age of the participants for a uveitis population, and the limited availability of clinical data. In this study we have considered measurements taken at a single visit, and therefore have not considered the stability of the signal over time.

Overall, our study shows that automated determination of VI is reliable across OCT B-scans in uveitis patients. The recommended minimum B-scan density for future research based on this parameter is three horizontal scans: One central scan and two peripheral scans located approximately 2000 µm inferiorly and superiorly from the central B-scan (3w). Measurements were even more stable across scans in a pattern of 7 horizontal scans (7c) but we interpret this improvement as not clinically relevant compared to the recommended pattern. In the future, further correlation of the data with clinical vitreous haze scores and other clinical variables as well as further reliability analysis based on these values is warranted.

## Methods

The retrospective study took place at the department of ophthalmology of the University of Bonn, Germany. The institutional Ethics Committee (University Hospital Bonn, Germany) approved the use of retrospective data for study purposes and approved that informed consent can be waived due to the use of retrospective data only (no. 103/18). The study adhered to the principles of the declaration of Helsinki. Participants were included if they had a form of Posterior Segment Involving Uveitis (PSIU) i.e. one of Intermediate, posterior or panuveitis as classified according to the SUN criteria^3^.

### Image data

Macular OCT volume scans were retrospectively collected. OCT is a light-based, non-invasive technique frequently applied in ophthalmology. It is based on local interference between two signals (object signal and reference signal)^24,25^. Using software, B-scans (e.g. axial) are automatically calculated from A-scans. Retinal OCT B- scans show parts of the posterior vitreous cavity, the retinal layers as well as choroidal structures. The volume scans were obtained with the Spectralis SD-OCT (Heidelberg Engineering, Heidelberg, Germany), with a B-scan image resolution of 512 × 496 pixels and 5 images averaged (automated real-time tracking mode = 5). Inclusion criteria were volume scans consisting of 19 B-scans each and a B-scan size of 20° × 15°. Exclusion criteria were insufficient image quality (HEYEX software image quality score < 20 in > 3 B-scans), incomplete scan, fixation errors and a presumed disease aetiology other than uveitis. Besides image data, age, uveitis classification, best-corrected visual acuity (BCVA) and lens status of all included patients were collected.

### Image analysis

Every other B-scan was selected from the OCT volume scans, resulting in 9 B-scans per volume scan available for analysis (Fig. 1). As one of the previous VI algorithm validation studies included a reference of 7 B-scans per volume, we used a comparable density as our gold standard^11^. The image data and additional image acquisition parameters were imported into MATLAB, Version R2016a (The MathWorks, Natick, Massachusetts, USA). The VI parameter Vitreous/Retinal Pigment Epithelium (RPE)-relative intensity was automatically calculated per B-scan according to an algorithm that has previously been described and clinically validated^8–12,26^. In summary, pre-processing steps include opening, thresholding and adjustment as outlined by Keane et al.^9^. The posterior part of the vitreous cavity is automatically detected and the OCT sum signal in this area is quantified relative to the RPE signal intensity in order to lower the impact of media opacities on the outcome parameter. The overall vitreous reflectivity is increased in inflammation which has been explained e.g. by inflammatory components and proteins in the vitreous cavity^9,26^. B-scan quality was assessed for all selected B-scans and the distance between B-scans was obtained per individual volume scan.

### Statistical analyses

The intra-class correlation coefficient between all VI values per volume scan was calculated. The single VI value of the central B-scan (1c) and averaged VI values of five combinations of B-scans (3, 5 and 7 central scans (3c, 5c, 7c), all 9 scans available for analysis (9 s), 3 widely distributed scans (3w); Fig. 1) were computed for all volume scans included. The averaged VI value of 9 B-scans was used as the standard reference. Mean absolute differences between this reference and a single central B-scan VI as well as the averaged VI values listed above (3c, 5c, 7c, 3w) were calculated. 95% limits of agreement (LoA) were calculated according to the formula LoA = mean ± 1.96 × standard deviation of the differences between the two measurements. Linear regression analysis was performed based on Bland–Altman plots to identify associations between the above mentioned mean absolute differences (e.g. 9 s-1c, 9 s-3c, 9 s-5c, etc.) and their respective means, excluding four cases that were likely outside of the sensitivity range of our study (mean VI score > 0.1). In addition, we performed receiver operating curve characteristic (ROC) analysis for discrimination of VI values greater or equal and VI values smaller than the median VI value out of all B-scans.

Statistical analyses were performed with SPSS Statistics, version 25 (IBM Corporation, Armonk, New York, USA) and R, version 3.5.0 (R Core Team, Vienna, Austria). Paired samples were compared with the Wilcoxon rank sum test correcting for multiple comparisons using the Holm-Bonferroni method^27^. The level of statistical significance was *P* < 0.05.
